# Malignant Ossifying Fibromyxoid Tumor of the Chest Wall Metastasized to the Lung Even After Complete Resectioning of the Primary Tumor - A Case Report and a Comprehensive Review

**DOI:** 10.7759/cureus.44793

**Published:** 2023-09-06

**Authors:** Harsh Patel, Vedant Shah, Neel Patel, Amy Tatsas, Saketh Palasamudram Shekar

**Affiliations:** 1 Clinical Research Management, Rutgers University, Newark, USA; 2 Internal Medicine, Nathiba Hargovandas Lakhmichand (NHL) Municipal Medical College, Ahmedabad, IND; 3 Public Health, Icahn School of Medicine at Mount Sinai, New York City, USA; 4 Pathology, Pathology Associates, Huntsville, USA; 5 Pulmonary and Critical Care, Pulmonary and Sleep Associates, Huntsville, USA; 6 Pulmonary and Critical Care, University of Alabama at Birmingham School of Medicine, Huntsville, USA

**Keywords:** clinical pathology, case report, evidence-based medicine, oncology, pulmonology, pulmonary metastasis, malignant ossifying fibromyxoid tumor of the chest wall, ossifying fibromyxoid tumor

## Abstract

An ossifying fibromyxoid tumor is a soft tissue neoplasm with ambiguous differentiation and low metastatic potential. Most cases involve the lower extremities, followed by the trunk, the upper extremities, and the head and neck region. It mainly arises in 40-70 years of age, and men dominate the disease's gender distribution. In the described types of ossifying fibromyxoid tumors, there are three variants: one is benign (typical), the second is malignant, which carries the risk of disease recurrence or metastases, and the third is atypical, which does not meet the criteria of either typical or malignant.

Here, we present an interesting case of a malignant ossifying fibromyxoid tumor of the chest wall that metastasized to the lungs even after complete resection of the primary tumor. A 64-year-old man had a 4.0 cm malignant ossifying fibromyxoid tumor in his chest wall two years ago, and at that time, the tumor was removed entirely. On pathology review, it was noted to have 20 mitotic figures per 50 high-power fields, but no actual grade was given. He was given postoperative radiation. His recent computed tomography (CT) chest with contrast showed a new right upper lung lobe nodule measuring 0.78 cm compared to the previous contrast-enhanced CT chest six months ago. It was worrisome for metastasis. F-18 FDG positron emission tomography scan revealed sub-centimetric pulmonary nodules in the right upper lobe. Right upper lobe lung biopsy showed spindle cell neoplasm morphologically consistent with the patient's known history of malignant ossifying fibromyxoid tumor. Biopsy demonstrated fragments of the bronchial wall and alveolated lung parenchyma with a focal spindle cell proliferation demonstrating a fibromyxoid matrix. The patient was referred to the oncologist for further management.

In conclusion, aggressive malignant ossifying fibromyxoid tumors can be found in atypical locations, e.g., the chest wall. Therefore, early diagnosis is crucial because of the high chances of metastasis to distant organs (including the lung) even after complete resection of the primary tumor. Even in asymptomatic patients, it is necessary to complete long-term follow-up for recurrence and metastasis surveillance of ossifying fibromyxoid tumors. Early recognition of recurrence or metastasis can decrease morbidity and mortality and improve overall organ function and patient survival.

## Introduction

Ossifying fibromyxoid tumor (OFMT) is a rare soft tissue tumor. It most commonly arises in the subcutaneous tissues of the trunk or extremities. Occasionally, it can involve the head and neck [1]. It is typically a painless, well-circumscribed tumor. Initially, it was believed to be benign, but in some cases, it was found to be malignant [2, 3].

OFMT tumors that show malignant or atypical features are called malignant OFMT [4]. There is no universally accepted risk stratification. Some features associated with malignant or metastatic potential are high cellularity, a high nuclear grade, and a high mitotic count of more than two mitoses per 50 hpf [3, 5].

The histogenesis of these tumors is controversial. These tumors exhibit characteristic imaging findings and a broad spectrum of histopathologic features, depending on the subtypes of the tumor - typical, atypical, or malignant. There are few cases of local recurrences. and rare cases of distant metastases. Complete tumor resection is the preferred treatment for these tumors, and a long-term follow-up is required as these tumors have an uncertain malignant potential [6]. This article presents a fascinating case of a malignant OFMT of the chest wall that metastasized to the lungs even after removing the primary lesion and radiotherapy.

## Case presentation

A 64-year-old male patient with a past medical history of a malignant ossifying fibromyxoid tumor of the chest wall presented to the oncology outpatient office after finding a new right upper lung nodule measuring 0.78 cm on the computed tomography chest with contrast. His previous computed tomography chest was normal six months ago. However, the previous lesion was 4.0 cm in size, was located on the right paramedian chest wall, peripheral to the sternum, and had been resected with a positive posterior margin two years ago. Subsequent surgery was performed to remove the deep positive margins. Post-operative radiation therapy was completed with 66 Gy delivered in 33 fractions via an image-guided volumetric modulated arc therapy (IG-VMAT) plan. Pathology revealed 20 mitotic figures per 50 high-power fields, but no grade was given. On the current visit, the patient had some sensitivity in his chest around his surgical site; otherwise, he was feeling well and asymptomatic. The patient has been followed up periodically after diagnosis and resection of the primary tumor. No local recurrence was detected.

**Figure 1:**
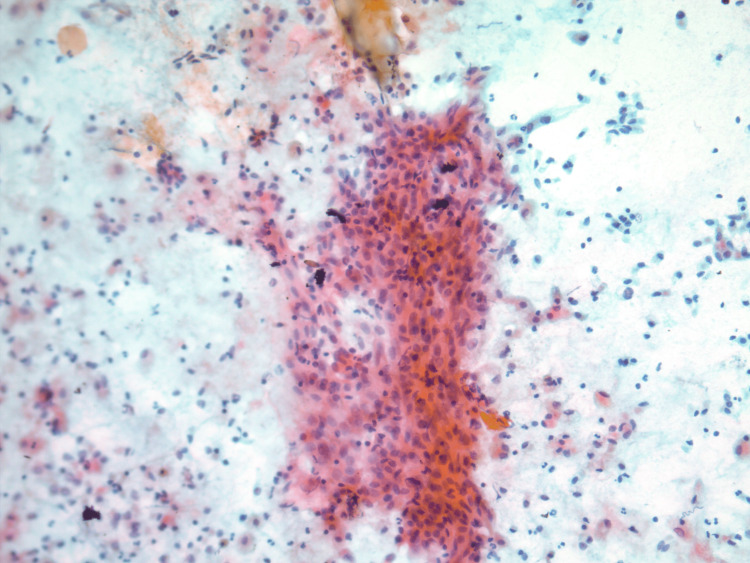
Papanicolaou (PAP) stained cytospin preparation demonstrates bland spindle cells in a background of few inflammatory cells

**Figure 2:**
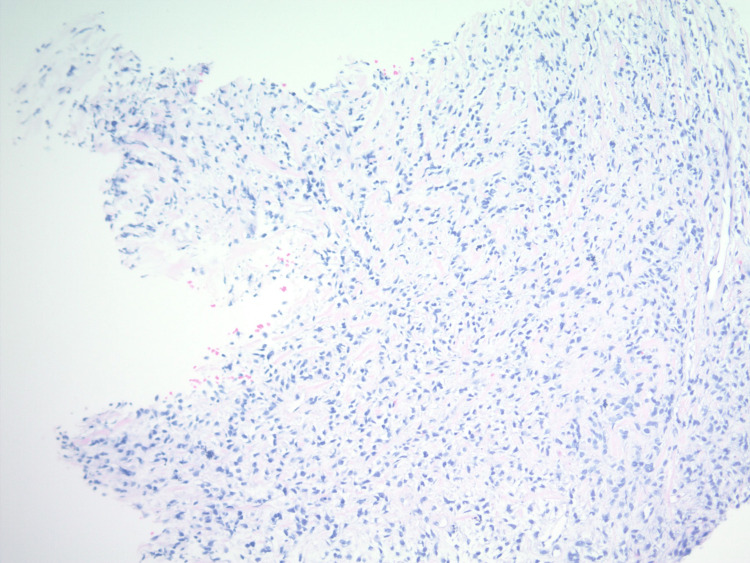
H&E stained sections at 100x show a bland spindle cell proliferation with a fibromyxoid matrix, consistent with the history of malignant ossifying fibromyxoid tumor

**Figure 3:**
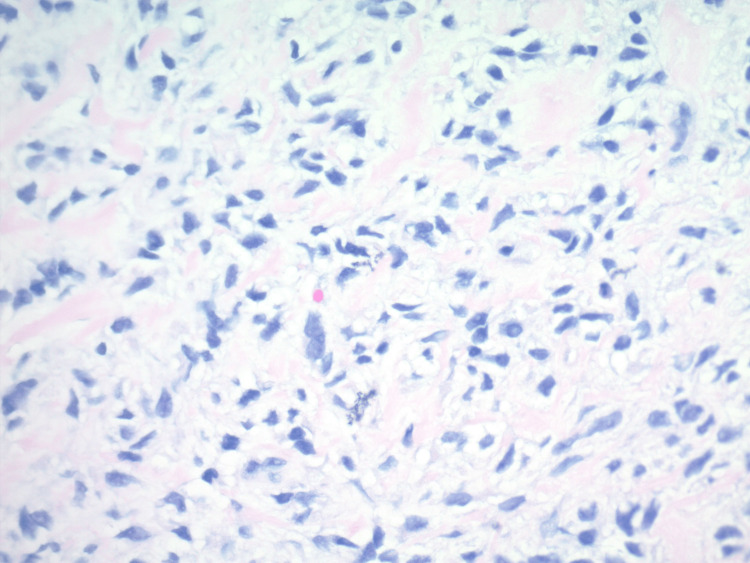
H&E stained sections at 400x show a bland spindle cell proliferation with a fibromyxoid matrix, consistent with the history of malignant ossifying fibromyxoid tumor

**Figure 4:**
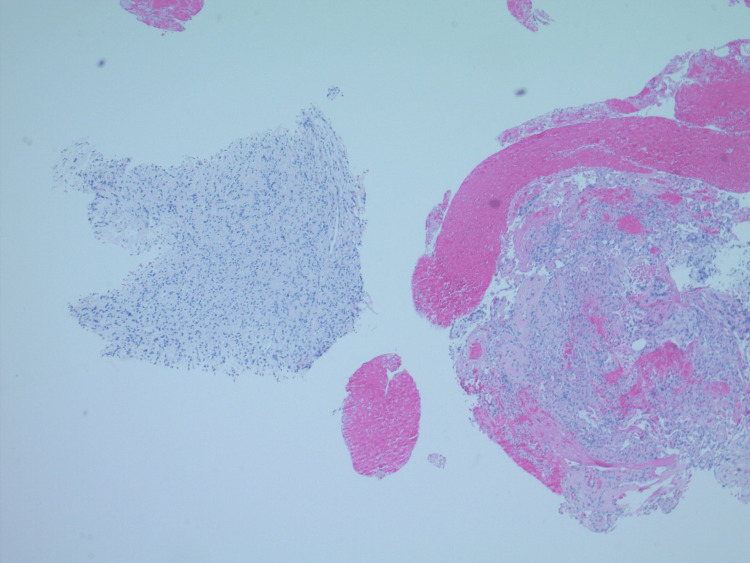
20x, benign lung parenchyma is seen on the right side of the field for reference

Due to the recent development of the new nodule on the CT chest, the case was presented at a multidisciplinary chest conference at the hospital for further management, and the team decided to proceed with a bronchoscopic biopsy and positron emission tomography scan. An F-18 FDG positron emission tomography scan revealed sub-centimetric pulmonary nodules in the right upper lobe that were not significantly FDG avid but were likely below PET resolution. It also showed ill-defined soft tissue density along the chest with mild generalized activity, suspected to be postsurgical in nature, given the history of resected chest mass in this location. A navigational bronchoscopy with a right upper lobe lung biopsy confirmed bronchial wall fragments and alveolated lung parenchyma with focal spindle cell proliferation, demonstrating a fibromyxoid matrix (Figures 1, 2, 3, and 4). Thus, the biopsy showed a spindle cell neoplasm morphologically consistent with the patient's known history of malignant ossifying fibromyxoid tumor. Trans-bronchial needle aspiration (TBNA) of the right upper lobe nodule demonstrated spindle cell neoplasm. Right upper lobe bronchoalveolar lavage (BAL) and brush tip showed benign respiratory epithelial cells and few pulmonary alveolar macrophages. Few atypical cells were present, and the Grocott methenamine silver (GMS) stain was negative for fungal organisms.

The patient was diagnosed with pulmonary metastasis of his resected primary tumor, ossifying fibromyxoid tumor of the chest wall. The patient was referred to an oncologist for further management.

## Discussion

OFMTs were first described by Enzinger et al. in 1989 [7]. OFMT usually affects the young, middle-aged, and elderly [8]. OFMTs are found 1.5 times more frequently in men than women [9]. The most frequent locations of OFMTs are the proximal limbs and limb girdles, distal upper and lower limbs, head and neck, and trunk. Although they often originate in the subcutaneous tissues, they can also affect deep muscle and bone sites [8]. In our case, the tumor originated from the subcutaneous tissues of the paramedian chest wall and spread to the lungs.

OFMTs are classified as typical, atypical, or malignant OFMTs based on characteristics such as nuclear grade, cellularity, and mitotic activity. The incidence of malignant OFMT is scarce, and few articles have been published regarding the metastasis of the primary OFMT to the lungs. Malignant tumors have a high nuclear grade, high cellularity, and mitotic activity of more than two mitoses per 50 hpf. Atypical tumors, however, do not meet all criteria [5]. Even though typical and atypical tumors are not considered malignant, they can recur (12% and 13%, respectively) and spread to other body parts (4% and 6%, respectively). On the other hand, malignant OFMTs exhibit a significantly higher risk of local recurrence and distant metastasis (60% and 60%, respectively) [5]. Upon diagnosing the disease two years ago, the malignant OFMT case discussed in our report demonstrated a high nuclear grade, high cellularity, and high mitotic activity of 3 mitoses per 50 hpf.

It is currently unknown what causes an OFMT and exactly how it differentiates. The most crucial aspect of OFMTs is their lineage of differentiation. They were traditionally believed to be derived from cartilaginous or schwannoma origins [10,11]. Graham et al. suggested that OFMTs may have a neuronal lineage of differentiation based on gene expression and proteomics data. Approximately 80% of OFMTs express excitatory amino acid transporter 4 (EAAT4), a member of the neuronal glutamic acid transporter family, which is highly expressed in the cerebral cortex [10]. In our case, EAAT4 staining was not performed, so the exact line of differentiation is unknown.

Microscopic findings of OFMTs are partially lobulated tumors with small, round cells with vesicular nuclei and indistinct cytoplasm. These cells are arranged in a cord- or nest-like pattern within a myxoid matrix, frequently displaying transitions toward hyaline fibrosis and focal osteoid formation. The incomplete shell of mature bone in the tumor's capsular region can be seen [7,12]. In our case, a right upper lung nodule biopsy showed a similar picture consistent with OFMT and the presence of bronchial fragments and alveolated lung parenchyma.

OFMTs resemble other mesenchymal tumors microscopically; therefore, immunostaining for molecular markers is essential for diagnosis [13]. The peripheral rim of bone tissue and positive S-100 immunostaining are the best predictors of typical OFMTs. Up to 20% of OFMTs are classified as non-ossifying OFMTs because they lack ossifying tissue [14]. Myoepithelial neoplasms, epithelioid nerve sheath tumors, low-grade fibromyxoid sarcomas, and sclerosing epithelioid fibrosarcomas are among the differential diagnoses for OFMT based on histomorphologic findings [8].

Both typical and malignant OFMT had very similar gene expression profile patterns (e.g., the elevation of EAAT4 and MUC4 and downregulation of peripheral myelin protein 22 (PMP22) and myelin expression factor 2 (MYEF2)) compared to nerve sheath myxoma/schwannoma, with no significant differences when typical and malignant OFMT were directly compared. Immunohistochemically, typical and malignant OFMT exhibited nearly comparable frequencies of expression of the different markers evaluated, with malignant OFMT showing lower expression of S100 protein. Reduced S100 protein expression in malignant OFMT likely signifies the progression of the neoplastic cells [10].

Folpe et al. suggested that the grade and proliferation of the tumors are significantly correlated with the recurrence and distant metastasis rates of OFMT [5]. However, Miettinen et al. could not identify any metastasizing OFMTs and stated that these metastatic tumors could be undiagnosed or misdiagnosed malignancies like sarcomas [9]. In our case, a lung nodule biopsy showed a morphologically consistent picture similar to the previous chest wall mass.

The tumor's behavior determines the treatment of OFMTs [9]. Typical OFMTs are treated with a conservative approach involving complete excision with wide margins [9]. There is limited literature regarding adjuvant radiation and chemotherapy in aggressive OFMTs. Our literature review found two OFMT cases where radiation therapy was used to treat the tumor and prevent local recurrence [13,15]. In these case reports, after using adjuvant radiation therapy, one patient was disease-free for more than 5 ½ years after resection, and the second was disease-free for more than 18 months on the routine follow-up. In our case, radiation therapy was also used postoperatively to prevent a recurrence. Table 1 describes previously published studies.

**Table 1:** Previously published case reports on metastasized ossifying fibromyxoid tumors of the lung F - female, M - male, OFMT - ossifying fibromyxoid tumor, LLL - left lower lobe, RLL - right lower lobe

Author name (Year)	Age (years)/ sex (male or female)	OFMT primary tumor location	Organs involved in metastasis	Time between primary tumor and metastasis	Signs and symptoms	Treatment	Outcome
Lastra et al. (2014) [16]	55/F	Left ankle	Lungs - large mass in the LLL, pleural effusion, three small nodules; thyroid - multiple masses with distinct firm nodules	12 years	Left chest and upper shoulder pain	Left below-knee amputation, resection of the LLL lung, right hemithyroidectomy, and isthmusectomy	Close follow-up for 12 years with no evidence of recurrence, then metastasis was detected
Nishio et al. (2002) [17]	52/M	Right foot	Lungs - multiple metastasis; thigh - multiple metastasis; right kidney; right gluteus maximus muscle	3-4 years	Asymptomatic primary mass	Surgical resection of the primary tumor, pulmonary lesions, and thigh, nephrectomy, chemotherapy (doxorubicin, ifosfamide, and dacarbazine)	Died due to widespread metastasis 5 years after the initial diagnosis
Suehiro et el. (2006) [2]	38/F	Skin lesion on scalp	Upper mediastinum and upper half of the right hemithorax (with mechanical compression of the superior vena cava, right lung, and trachea); brain; lungs - multiple metastases	6 years	wheeze, productive cough, signs of superior vena cava obstruction	Excision of primary skin lesion, and the mediastinal mass (including most part of the right lateral pericardium with right phrenic nerve, pulmonary artery, both veins, and the whole right lung), radiotherapy	Readmitted with seizures due to multiple brain metastases at 5 months, multiple lung metastases in the opposite lung at 6 months, and died 8 months after initial surgery
Schaffler et al. (1997) [18]	32/M	Left side of back at the level of the eighth thoracic segment	Extensive local recurrences; anterior abdominal wall; lungs - multiple metastases, malignant pleural effusion	2 years	-	Resection of the primary mass	Death due to tumor
Sarraj et al. (2007) [19]	71/M	Left leg	Lungs - LLL mass extending through the left inferior pulmonary vein and protruding into the left atrium and left ventricle; heart - large mass in the posterior region of the left atrium; bone - proximal fibula	-	dyspnea, orthopnea, signs of heart failure, paroxysmal atrial tachycardia	Resection of primary tumor in left leg, tumor resection through left atriotomy using cardiopulmonary bypass and cardiac arrest, and left lower lobectomy of the lung	No local recurrence at follow-up
Sbaraglia et al. (2020) [20]	65/F	Rib	Lungs - 3 cm lesion of the LLL; recurrence - 5 cm tumor in the right rib	5 years	-	Resection of the primary tumor, atypical lung resection, no systemic treatment	No local recurrence at follow-up
Minami et al. (2001) [21]	70/M	Right buttock	Lungs - multiple small nodules peripherally	At the time of the diagnosis	-	Total excision of the tumor with the gluteus maximus muscle	Enlarging pulmonary nodules without a local recurrence at 10 months follow-up
Kilpatrick et al. (1995) [12]	68/M	Greater trochanter of the right femur	Lung - 8 mm pulmonary nodule in RLL	-	Right hip pain	Local excision of right hip mass	Enlargement of the nodule with new second nodule in the lung several months later, local recurrence, and additional pulmonary metastasis (both were excised) 25 months later

## Conclusions

Aggressive malignant ossifying fibromyxoid tumors can be found in atypical locations, e.g., chest wall. Therefore, early diagnosis is crucial because of the high chances of metastasis to distant organs (including the lung) even after complete resection of the primary tumor. Even in asymptomatic patients, it is necessary to close long-term follow-up for recurrence or metastasis surveillance of ossifying fibromyxoid tumors. Early recognition of local recurrence or metastasis can decrease morbidity and mortality and improve overall organ function and patient survival.
